# Bibliometric Analysis of Functional Crops and Nutritional Quality: Identification of Gene Resources to Improve Crop Nutritional Quality through Gene Editing Technology

**DOI:** 10.3390/nu15020373

**Published:** 2023-01-11

**Authors:** Xun Wei, Yan Long, Chenchen Yi, Aqing Pu, Quancan Hou, Chang Liu, Yilin Jiang, Suowei Wu, Xiangyuan Wan

**Affiliations:** 1 Zhongzhi International Institute of Agricultural Biosciences, Shunde Innovation School, Research Center of Biology and Agriculture, University of Science and Technology Beijing, Beijing 100024, China; 2 Beijing Beike Institute of Precision Medicine and Health Technology, Beijing 100192, China

**Keywords:** gene editing, functional crops, nutritional quality, bibliometric, hidden hunger, food security

## Abstract

Food security and hidden hunger are two worldwide serious and complex challenges nowadays. As one of the newly emerged technologies, gene editing technology and its application to crop improvement offers the possibility to relieve the pressure of food security and nutrient needs. In this paper, we analyzed the research status of quality improvement based on gene editing using four major crops, including rice, soybean, maize, and wheat, through a bibliometric analysis. The research hotspots now focus on the regulatory network of related traits, quite different from the technical improvements to gene editing in the early stage, while the trends in deregulation in gene-edited crops have accelerated related research. Then, we mined quality-related genes that can be edited to develop functional crops, including 16 genes related to starch, 15 to lipids, 14 to proteins, and 15 to other functional components. These findings will provide useful reference information and gene resources for the improvement of functional crops and nutritional quality based on gene editing technology.

## 1. Introduction

Among the global population of 8 billion, hunger has affected 828 million in 2021 and is expected to increase continuously [1]. In addition to the population pressure, climate change and environmental shocks are reducing food production, making global food security even more critical and complex. According to a report released by the United Nations Children’s Fund (UNICEF), about 340 million children under the age of five all over the world are suffering from “hidden hunger”, which refers to hidden nutritional needs due to nutritional imbalances or deficiencies in certain micronutrients and essential minerals. At the same time, in pace with economic development, there is a higher and stronger demand for eating better and healthier; the needs for functional foods and their important raw materials or functional crops have grown accordingly [2]. Thus, besides the goals of high yields, high resistance, and efficient utilization of nutrients in the production of major crops, improving nutritional and functional quality has become the focus of crop breeding.

Currently, many kinds of key functional components of functional foods come from crops. In recent years, soybean oligosaccharides extracted from the by-products of soybean protein concentrates have been widely used in food and pharmaceutical industries. It not only plays a role in delaying starch aging, but also is fully utilized by *Bifidobacterium* to promote the reproduction of *Bifidobacterium* in the intestine, increase intestinal motility, and regulate blood lipids and blood sugar [3]. Bioactive peptides, which are obtained by concentrating proteins in crops, can be added to foods or nutritional health products. Soy protein peptides are natural nutrients obtained from soybeans and can be made into intestinal nutrients to provide complete nutrition for people who cannot eat conventional foods [4]. In 1996, the Food and Drug Administration (FDA) of the United States approved soybean isoflavones as functional foods to go on the market for the prevention of cardiovascular disease and to improve osteoporosis in women [5]. Collectively, it is important to improve the main nutritional ingredients in crops for developing functional foods.

The cultivation of functional crops through biotechnology mainly depends on the means of genetic engineering, including gene overexpression and gene editing [6]. At present, gene editing has been widely used in crop breeding, especially that targeting quality-related traits. The content and structure of starch, proteins, and lipids are the main indexes affecting the quality of crops. Through gene editing technology, crop varieties have been improved in starch content, aroma, nutritional value, and storage quality. Ma et al. knocked out the rice *Wx* gene through CRISPR/Cas9 technology, reducing rice amylose content from 14.6% to 2.6% [7]. Achary et al. used CRISPR/Cas9 to mutate the rice *GW2* gene, improving the nutritional quality of the rice aleurone layer and grains [8]. The CRISPR/Cas9 system was also used to improve the ratio of 7S protein and 11S protein in soybean [9]. Liang et al. successfully reduced the phytic acid content in corn by using CRISPR/Cas9, which improved the nutritional value of maize [10]. Functional components in wheat, rice, corn, and other crop varieties, such as alkyl resorcinol, β-glucan, and flavonoids, have been proven to possess many bioactivities based on in vitro animal models and health effect evaluations. Zhu et al. obtained red rice rich in anthocyanins by using gene editing technology, CRISPR/Cas9, to mutate the rice *Rc* gene at a fixed point [11]. However, most crops that are improved by gene editing are still only available in the laboratory [12], and there are only six types of gene-edited crops that have been planted commercially [13]. Besides the concern of the maturity and cost of gene editing technology, such as PAM sequence limitations in CRISPR/Cas technology and low genetic transformation efficiency in some plants [14,15], the most important impediment of its application is the strict policy environments, especially in some European countries and China [16]. However, with the deregulation of gene-editing technology in many countries, the commercialization of gene-edited crops will be accelerated [17,18].

Mining potential genes plays an important role in improving crop quality, breeding functional crops, and further developing functional foods. This study analyzes the research hotspots and trends in the field of functional crop improvement using gene editing technology based on bibliometrics. Then, we comprehensively summarize the gene resources that can be used to improve crop quality via gene editing. This is of great significance for researchers to further improve the beneficial components of functional crops and develop functional high-quality crops with nutrition to alleviate the food security crisis and hidden hunger.

## 2. Methods

### 2.1. Bibliometric Analysis

Bibliometric analysis is an effective method for a quantitative and comprehensive review of a certain scientific area, with two main means of performance analysis and science mapping [19]. The performance analysis of bibliometrics is based on the literature database and visual bibliometric analysis software, including authors, institutions, national cooperative analysis, literature citation, co-citation analysis, cluster analysis of keywords and hot words, etc. The scientific atlas is the transformation of a bibliometric performance analysis into a more intuitive and vivid visual analysis network graph with the help of visual bibliometric analysis software [20].

In this study, publications were searched from the core collection of Science Citation Index Expanded (SCI-E) in the Web of Science on 24 September 2022. The search strategy was based on keywords related to gene editing technology, various crops, and main nutrient components (see Table S1). The time period ranged from 2005 to 2022, and the literature types were selected as articles or reviews. From the title and abstract, the literature with a poor correlation with the research topic was excluded, and 485 effective and high-quality documents were retained from the search results as the data source for this study. The abstract, complete records (including references), and other information retrieved from the Web of science core database were converted into the form of reference and exported as pure text files to establish the literature database. Next, the database was imported as text into the visual bibliometric software for visual analysis. In this study, bibliometric analysis was carried out using CiteSpace 6.1.R6 (Drexel University, Philadelphia, PA, USA) [21]. From each import file, the interactions and interrelationships among authors, journals, and countries were revealed, as well as a visual analysis of the evolution of the keywords, literature co-citation, co-occurrence, and co-citation, which helps facilitate an understanding and analysis of the current situation of literature research in this field and provides a more comprehensive and systematic field research review for scholars conducting related research.

### 2.2. Literature Review

The purpose of the literature review through bibliometrics was to explore the potential genes for functional crop breeding. In order to improve the application of gene editing technology in terms of the functional components of crops, we included articles on the mining and identification of gene resources of carbohydrates, functional lipids, proteins, flavonoids, minerals, vitamins, and other active substances in rice, wheat, corn, and soybean. This review included screening the abstracts of 485 papers, and 53 papers related to this study were finally selected as appropriate.

To increase reliability, the papers were read and screened by two to four people. The contents of the papers were summarized in a database, taking into account the research purpose, significance, methods, categories of crops, technical means, nutritional functions and other factors. Different views and opinions on individual papers were resolved in the discussion. The database provides research on gene editing, functional components of crops, and health benefits in humans.

## 3. Results

### 3.1. Publication Volume and Distribution within Crop Varieties and Functional Components

**Figure 1:**
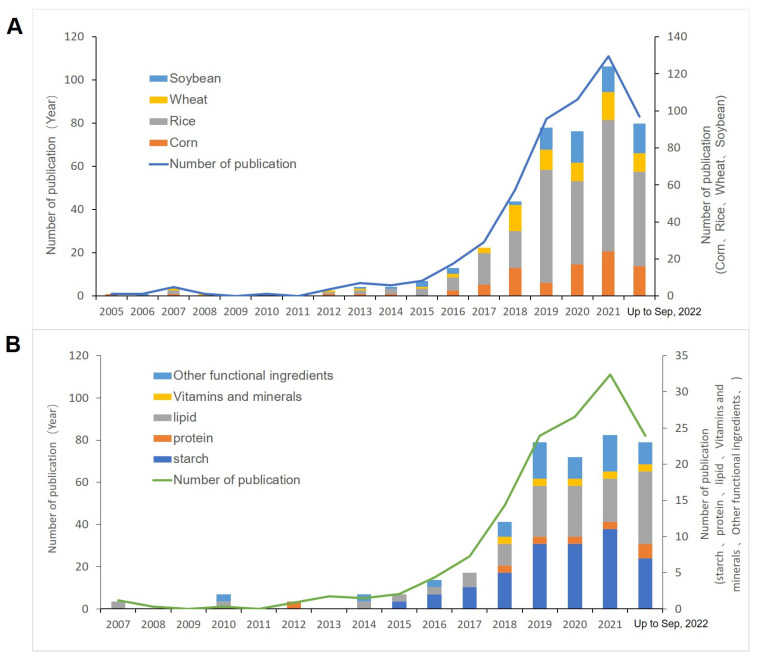
Number of publications and information on gene editing to improve crop quality and function. (**A**) Number of publications in the field of gene editing for improving crops based on corn, rice, wheat, and soybean from 2005 to 2022; (**B**) number of publications in the field of gene editing for improving crops using maize, rice, wheat, and soybean from 2005 to 2022.

When we traced the research history, it was found that the relevant literature was first published in 2005, and the publication volume did not significantly increase until 2015 (Figure 1). The sudden increase in the publication volume around 2015 is most likely due to major breakthroughs in relevant technologies and their application with the support of policy environments. In 2013, Zhang et al. used CRISPR/Cas9 for genome editing in eukaryotic cells [22], and Gao et al. published an article on CRISPR/Cas9 editing in plants for the first time [23]. These studies initiated the development of gene editing in the field of crop improvement. In 2016, Li et al. proposed a framework for the management of genome-edited crops, presenting the idea of treating genome-edited products similar to conventional breeding products [24]. Using the CRISPR/Cas9 multiplex genome editing method, Shen et al. created a vector that targets eight rice agronomic genes. They discovered that the eight target genes exhibit high mutation efficiencies in the T-0 generation through further genetic modification and DNA sequencing. All editing-associated genes underwent both heterozygous and homozygous mutations in T-0 plants. Numerous phenotypes connected to the editing-associated genes were seen in T-0 transgenic plants as a result of their numerous genotypes. Their findings showed the potential of the CRISPR/Cas9 system for quickly introducing genetic variation during crop breeding [25,26]. In 2018, Appels, R et al. published the final results of sequencing of the hexaploid wheat genome. Therefore, gene editing research on wheat has increased and has remained in a stable state so far.

Among four of the world’s most economically important food crops, rice is the most researched crop, followed by maize (Figure 1A). Relatively little research has been performed on soybean and wheat. This may be because rice is a diploid species, with the smallest genome of the four crops, and has also been used as a model grain for molecular studies [27], while maize has higher cost-effectiveness. According to the statistics of starch, protein, lipids, vitamins, minerals, and other functional components in crops (Figure 1B), it was found that lipids were the most frequently studied in this field, followed by starch, and the publication volume of related studies started to surge in 2018 and is still gradually increasing. The proportion of the publication volume of each component has remained essentially the same.

### 3.2. Map of Cooperation and Competition among Countries

**Figure 2:**
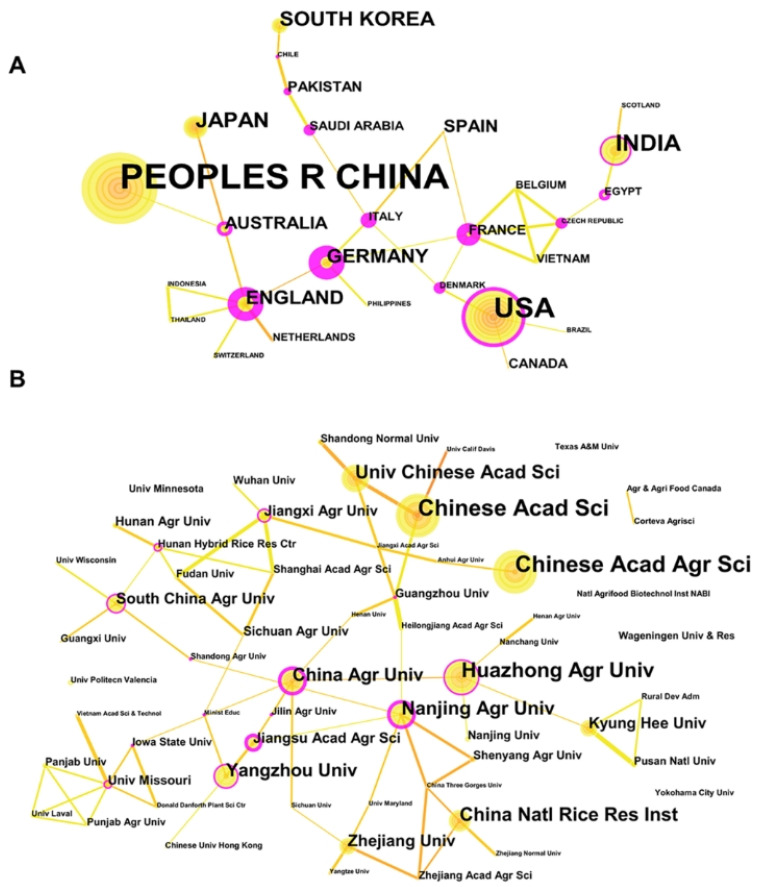
Country and institution cooperation and competition relationship map and journal distribution. (**A**) National cooperation network among 27 countries, reflecting the volume of publications by the size of the yellow circle and the intensity of cooperation by the thickness of the lines between the nodes; (**B**) cooperation network of 58 institutions.

The activity and impact of countries’ research can be reflected by the number, centrality, and citations of publications. As far as the centrality represented by the thickness of the red circle shown in Figure 2 is concerned, countries that cooperate closely and frequently with other countries are the United States, Germany, England, France, Italy, and Australia, etc., which serve as a bridge for cooperation networks (Figure S1A). In contrast, countries such as China, Japan, and South Korea have relatively little cooperation, despite their leading position in terms of publication volume. Taking citation volume into account (Figure S1B), the United States is in a leading position in the field of gene editing and crop improvement. In addition, France, England, Spain, and other countries have high citation volumes. However, the Latin American and African countries are relatively inactive due to the technology gap of gene editing. Compared to that with the technology innovation itself, the application of gene editing to crop breeding needs less widespread cooperation, reflected by little interaction among clusters of institutes (Figure 2B).

The United States has always been in a leading position in the field of bio-agriculture, and it is the country with the most approvals for genetically modified crops. Since 2012, the United States has successively agreed that *IPK1*-gene-edited corn using ZNF technology and cold storage tolerance *VInv* gene-edited potato using TALEN technology is not regulated by transgenic laws. The U.S. government has indicated that gene editing will not be classified as the regulation of transgenic crops because it will not introduce new genes, and thus, the government regulatory agencies are very relaxed in terms of the regulation of the whole gene editing field [28]. In 2015, the United States realized the commercial planting of SU Canola TM, a kind of rape, the world’s first genome-edited crop. Moreover, in 2016, the regulation of genetically edited crops was put forward to exempt genetically edited crops from supervision, which greatly promoted the development of genetically edited improved crops in the United States [29]. At present, the U.S. government has published some safety evaluation standards for genetically edited crops, such as the APHIS’ Biotechnology Regulations 7 CFR part 340 and others, for reference to other countries, which also illustrates the advanced nature of the U.S. in the field of gene editing and crop improvement from another angle [30]. The relatively well-established regulatory system of genetically edited crops in the United States has led to a very high level of motivation in the field of gene editing for crop improvement.

In 2018, Europe announced the classification of genetically edited crops as genetically modified crops [31]. Because its supervision of genetically modified crops has always been particularly strict and lagging, its restrictions on the use of gene editing are also very strict, even affecting the overall development of gene editing technology in Europe. The controversy over gene editing to improve crops in Germany has always been fierce. In 2015, the German National Academy of Sciences issued an opinion, hoping that the German government would review its transgenic regulations and redefine its regulatory scope. However, it has not received positive feedback from the German government. Fortunately, after the Nobel Prize in Chemistry was promulgated in 2020, the EU’s attitude towards gene editing has become somewhat relaxed. In September 2022, the results of the public consultation of the European Commission on “Legislation of Plants Produced by Some New Genome Technologies (NGTs)” showed that most people support the formulation of new policies to use new technology tools to promote sustainable agricultural development (ISAAA, 2022).

In recent years, scientists in China have published more articles on gene editing and crop improvement than any other country, but the number of citations is not high, which shows that the quality of articles published in China needs to be further improved. In January 2022, the Ministry of Agriculture and Rural Affairs of China formulated and published the Guidelines for Safety Evaluation of Genetically Edited Plants for Agriculture, indicating that China would begin to approve genetically edited crops, paving the way for the commercialization of genetically edited crops in China [32]. In May 2022, the release of the “14th Five-Year Plan for Bio-economic Development” added a more powerful impetus to the development of gene editing and crop improvement in China [33].

### 3.3. Research Hotspots and Trends

#### 3.3.1. Research Hotspots

**Figure 3:**
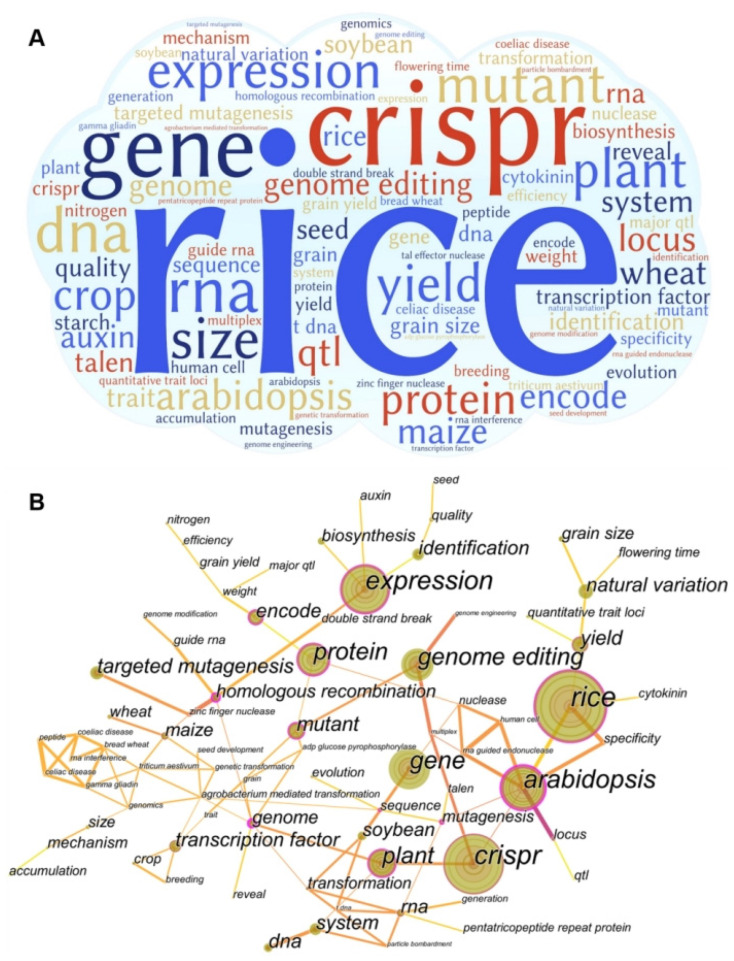
Research hotspots and trends. (**A**) Global gene editing research word cloud; (**B**) global gene editing research word cloud collinear diagram.

The analysis of keywords is explained according to the knowledge structure and hot topics in this field (Figure 3). Keywords are the core vocabulary, highly summarizing the literature content and providing concise expressions of the literature topics, and high-frequency keywords are often used to determine a hot issue in a research field. As can be seen from Figure 3A,B, the research keywords in the field of gene editing mainly focus on rice, CRISPR, gene, expression quantity, quality, protein, plant, wheat, soybean, corn, crops, etc. Among them, the keywords closely and frequently linked with other research focuses are *Arabidopsis thaliana*, rice, expression quantity, CRISPR, protein and gene editing, etc., as the bridge of the contact network. It can be further summarized from three aspects: the main research object, gene editing technology, and research direction.

As far as the research object is concerned, rice, corn, wheat, and rice are the most widely planted and important grain varieties in the world. The frequent occurrence of climate change, natural disasters, cultivated land reductions, and epidemic diseases has posed a serious threat to food security [34]; especially, the import of soybeans, corn, and other cereals in China has increased by a large margin in recent years [32]. Therefore, increasing the grain output, improving the nutritional value of crops, and enhancing the ability of crops to resist pests and diseases have become urgent challenges that people are facing now.

Facing the increasing world population, according to the United Nation’s forecast, by the end of this century, global grain production needs to be increased by half based on the current production, but traditional breeding methods have a long cycle and low efficiency, which makes it difficult to achieve this goal. In recent years, although genetically modified crops have brought great developments to the agricultural economy, the related risks still cannot be ignored. In contrast, gene editing technology has become a more popularly accepted means of breeding, and its importance in agriculture is more prominent. ZFNs and TALENs are the previous two generations of gene editing techniques. The former is costly and easily causes cytotoxicity when improperly used, while the latter is difficult to perform [35,36]. CRISPR has become an excellent means of gene editing in recent years because of its simplicity and high efficiency [37]. CRISPR/Cas9 is currently patented in the United States, but a team from the China Agricultural University developed Cas12i and Cas12j in 2019 and obtained the patent in 2021, breaking the monopoly of foreign countries on this technology and filling the gap of the gene editing toolbox [17,38].

#### 3.3.2. Research Trends

As far as the research direction is concerned, quality is the most important economic characteristic of crops. With the improvement of people’s living standards, people’s demand for crops has changed from increasing the crop yield to improving crop quality. In the past decades, protein deficiency was once considered the most urgent nutritional problem in the world [39], and thus, research on improving the content and quality of protein in crop breeding occurred earlier than that on other nutritional qualities in crops. Nowadays, with increasing attention paid to the nutritional quality of crops, there is more research on starch, oil, and other active ingredients in crops.

**Figure 4:**
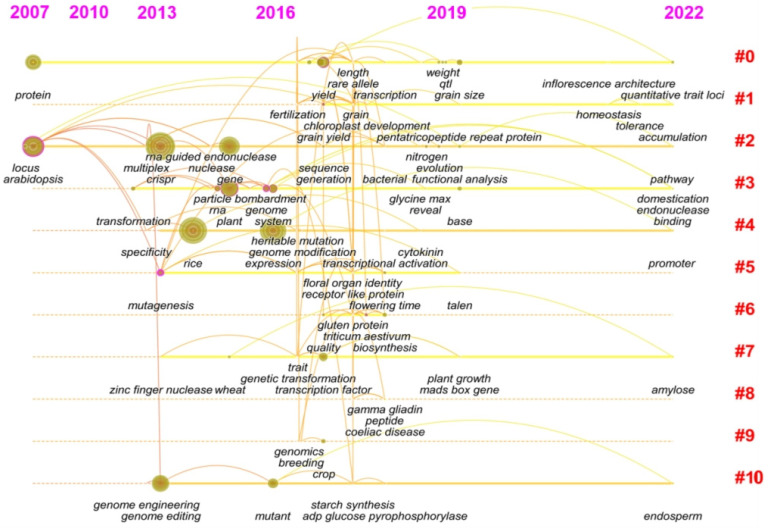
Timeline chart of keyword changes in the gene editing field from 2007 to 2021. Using the Timeline View clustering function in CiteSpace software, the dynamic frontier evolution map of keywords in the gene editing field was obtained (Figure 4). Based on text clustering analysis, 10 clusters were obtained, namely #0, rice; #1, chloroplast development; #2, functional redundancy; #3, cpf1; #4, homologous recombination; #5, regulatory network; #6, the main food crops; #7, wheat; #8, gluten; #9, economic value; and #10, endosperm development. This shows that the application of gene editing to food crops and gene editing technology has become the research focus in recent years. Moreover, the functional redundancy of rice shows the longest cycle from 2007 to 2021.

Since Doudna and Charpentier first used CRISPR/Cas9 in 2012 to achieve a major breakthrough in the field of gene editing [40], the field of gene editing has started to undergo a “blowout” development. Especially in plant breeding, “gene” and “heritable variation” became research hotspots from 2012 to 2016, and more research was performed on gene editing technology. Perhaps the most important event was that in 2015, Zhang et al. discovered a powerful substitute, the Cpf1 enzyme, which has different characteristics from Cas9 and improved ability to manipulate the eukaryotic genome [41]. In recent years, the research on genes and gene editing tools has turned to the specific application of gene editing tools, reflected by the keywords, such as amylose, accumulation, pathway, and gliadin, etc. (Figure 4 and Figure S2). The editing of genes related to the control of agronomic traits, nutritional quality, and enhancing the tolerance of major crops, such as rice and wheat, and research on regulatory networks and gene loci associated with quantitative traits in plants have drawn great research interest. After systematic evolution, the research hotspots now focus on the regulatory network of related traits, quite different from technical improvements in gene editing in the early stage.

## 4. Gene Resources and Strategies for Crop Nutrient and Quality Improvement

### 4.1. Genetic Resource Mining of Starch in Four Main Crops

Starch is the most important nutrient in crops and the main source of human energy, usually stored in the endosperm of crops as semi-crystalline starch granules. It accounts for about 70–80% of rice and over 74% of corn grains and 65–75% of the total dry weight of wheat grains [42]. As an oilseed crop, soybean is relatively low in starch, containing about 35% carbohydrates [43].

#### 4.1.1. Starch Types and Contents and Their Nutrimental Values in Crops

Starch is a carbohydrate consisting of linear amylose and highly branched amylopectin. The ratio of amylose to amylopectin determines the physicochemical property and nutritional value of starch-processing materials [44]. Amylose is beneficial to control blood sugar (diabetic condition) levels in diabetic patients due to its low glycemic index [45].

The concept of resistant starch (RS) was firstly proposed more than 40 years ago [46]. RS can resist the hydrolysis of amylase and is hardly digested in the small intestine. Due to the high level of digestive resistance, a diet rich in RS helps to reduce postprandial blood glucose levels and relieve insulin resistance in patients with diabetes. It has been proven that the content of RS is positively proportional to the content of amylose [47].

#### 4.1.2. Gene Resources for Starch Improvement in Rice, Corn, Wheat, and Soybean

Starch biosynthesis in plants begins with the conversion of glucose 1-phosphate to ADP glucose. The key enzymes of starch biosynthesis include ADP glucose pyrophosphorylase (AGPase), granule-bound starch synthase (GBSS), starch synthase (SS), starch branching enzyme (SBE), and starch debranching enzyme (SDE) [48].

SBE, which controls the synthesis of amylopectin, plays an important role in the synthesis of higher levels of RS in crops. Down-regulation or mutation of the gene encoding starch branching enzyme IIb (SBEIIb) leads to increased amylose content and RS levels in crops [49,50], thus further affecting the quality of starch. SBE changes the ratio of amylose and amylopectin in starch by affecting the content of amylopectin. The *Waxy* (*Wx*) gene encodes granule-bound starch synthase I (GBSSI), which has key effects on starch quality. Changing the activity of SBEII and GBSSI can regulate the amylose (AC) content in crops and obtain crops with high RS content, which are helpful to improve human health [51,52]. Many transcription factors (TFs) can also regulate the biosynthesis of starch. TFs mainly regulate cis-acting elements in target genes, such as NF-Ys [53], SPA [54], and bZIPs, which all act in the biosynthetic process of starch [55]. In conclusion, manipulating the genes of related enzymes in starch biosynthesis using gene editing, such as CRISPR/Cas9 technology, influences the content of amylose and RS and the ratio of amylose to amylopectin in crops and on this basis, can be used to produce functional crops with high quality and high nutrition or those that are beneficial to diabetics.

By exploring the genetic resources of rice, corn, and wheat, 16 key genes related to starch quality were obtained and confirmed using CRISPR/Cas9 gene editing (Table 1). For example, the knockout of *TaSBEIIa* (TraesCS2A02G293400) in wheat and *OsSBEIIb* (LOC_Os02g32660) in rice based on CRISPR/Cas9 gene editing technology resulted in increased amylose and RS content in wheat and rice, which are beneficial for patients with diabetes and kidney diseases [46,47,50,52,55]. The knockout of *Waxy* genes in wheat (TraesCS7A02G070100), corn (Zm00001eb378140), and rice (LOC_Os06g04200) leads to lower amylose content and higher amylopectin content and thus greatly improves the edible taste of wheat, corn, and rice [54,56,57,58,59]. These successful examples indicate that crop starch quality and nutrients can be greatly enhanced by manipulating the starch biosynthesis-related key genes based on gene editing technology.

### 4.2. Identification of Functional Lipid-Related Gene Resources in Crops

Lipids include saturated fatty acids and unsaturated fatty acids, among which the saturated fatty acids consist of palmitic acid and stearic acid, etc., and the unsaturated fatty acids are monounsaturated fatty acids, such as oleic acid, and polyunsaturated fatty acids, such as linoleic acid and linolenic acid. The proportion of fatty acid content in conventional soybean varieties is generally as follows: linoleic acid > oleic acid > palmitic acid > linolenic acid > stearic acid. Changing the fatty acid composition of soybean to improve the quality of soybean oil is an important and evolving theme. Corn oil and rice bran oil are two of the best edible vegetable oils published by the WHO [67]. Among them, corn oil is a by-product of the corn wet milling industry, which is extracted from the corn germ [68], mainly composed of 59% polyunsaturated (PUFA), 24% monounsaturated (MUFA), and 13% saturated fatty (SFA) acids. Rice bran oil (RBO) is the by-product of rice bran milling. Triglycerides (TAGs) account for about 85% of total lipids in RBO [69], and TAGs mainly consist of three kinds of fatty acids: palmitic acid (about 13–22%), oleic acid (about 37–52%) and linoleic acid (about 27–40%). The nutritional value of rice bran oil lies not only in its high content of unsaturated fatty acids, but also in its abundant tocopherol, γ-oryzanol, and other compounds with antioxidant and cholesterol-lowering activities [70]. In wheat, although lipids are also an important factor affecting the quality of wheat, they only account for 3–4% of the grain weight, and related comprehensive research is still lacking.

#### 4.2.1. Main Factors Affecting Crop Oil Quality

The composition and proportion of fatty acids determine the quality of seed oil. Saturated fatty acids and monounsaturated fatty acids can be synthesized in the human body, but some essential fatty acids needed to maintain the normal growth of the body cannot. The long-term intake of a large amount of saturated fatty acids tends to lead to hypertension and coronary heart disease. High levels of polyunsaturated fatty acids, such as linoleic acid or linolenic acid, are essential for a healthy diet, however, the reactive double bonds in linoleic acid and linolenic acid can be oxidized in situations without enzymes, thus shortening the shelf-life of seed oil, accelerating rice aging [71], and affecting rice quality. The trans-fatty acids produced via the hydrogenation of linoleic acid in vegetable oil can have adverse effects on the heart [72]. Omega-3 and omega-6 fatty acids are also polyunsaturated fatty acids, and studies have demonstrated that the balance of omega-3 and omega-6 in the diet is linked to risks related to coronary heart disease [73]. Oleic acid is called a “safe fatty acid” as it can reduce the total blood cholesterol and harmful cholesterol content, but not the beneficial cholesterol content [74]. Moreover, it can improve the stability and antioxidant capacity of oil and prevent the formation of trans fatty acids in vegetable oils by reducing hydrogenation [75]. Therefore, reducing the content of saturated fatty acids and increasing the content of unsaturated fatty acids are of great significance to improve the quality of crop oil.

Crops with a high fatty acid content generally have higher taste quality, and thus, improving the content of fatty acids in seeds and increasing crop nutrition is also a major focus of crop improvement.

#### 4.2.2. Gene Resources and Improvement of the Oil Quality in Rice and Soybean

Plant fatty acid synthesis primarily takes place in plastids, and these molecules are subsequently transported to the endoplasmic reticulum or other sites of the cytoplasm for processing into triacylglycerols [76]. Enzymes involved in fatty acid synthesis include acetyl-CoA carboxylase (ACCase) and fatty acid synthase (FAS). FAS is a multi-enzyme complex composed of an acyl carrier protein (ACP), β-ketoacyl-ACP synthase (KAS), β-ketoacyl-ACP reductase, β-hydroxyl-ACP dehydrase, β-enoyl-ACP dehydrase, enoyl-ACP reductase, and acyl-ACP thioesterase. The first step of fatty acid synthesis is malonyl-CoA formation from acetyl-CoA via acetyl-CoA carboxylase catalysis, and then, FAS takes malonyl-CoA as a substrate for a series of polymerization reactions to synthesize a carbon chain, and palmitic acid (C_16_) is synthesized via fatty acyl-ACP thioesterase catalysis [72]. The synthesis of saturated fatty acids, such as stearate (C_18_), should continue the extension of the carbon chain through KAS in smooth internal networks and mitochondria. Unsaturated fatty acids are synthesized via the desaturation of saturated fatty acids in microsomes, and stearic acid is the precursor of oleic acid synthesis. In plastids, stearoyl-ACP is desaturated by stearoyl ACP desaturase (SACPD) to obtain oil-based ACP, thus obtaining oleic acid. The monounsaturated fatty acids can be catalyzed by fatty acid desaturase 2 (FAD2) to produce linoleic acid [77].

Through mining the gene resources of rice and soybean, we have obtained 15 key genes related to improvements in the oil quality in crops based on gene editing technology (Table 2). For example, FAD2 is essential for controlling the content of oleic and linoleic acid in seed species, and several studies have shown that the inhibition of *FAD2* gene function based on gene editing leads to a significant increase in oleic acid content and a decrease in linoleic acid content in rice and soybean [77,78,79,80,81]. These examples suggest that crop oil quality and nutrients can be greatly enhanced through manipulation of the lipid biosynthesis-related key genes based on gene editing technology.

### 4.3. Identification of Functional Protein–Gene Resources in Crops

Seed protein is the main source of ingested protein for humans and livestock. It mainly includes structural proteins, protective proteins, and storage proteins, among which storage proteins are further divided into globulins, gliadins, and glutenin. Soybean is an indispensable raw material in animal feed due to its high protein content, high protein digestibility, balanced amino acid composition, availability, and relatively fair production cost. Generally speaking, soybean seeds contain 35–40% protein [88], with the storage proteins 11S globulin and 7S globulin accounting for 70–80% of the total protein [89]. The total protein content of rice seeds is only about 8%, which is one of the crops with the lowest protein content. The structural proteins in rice are very scarce, while the storage proteins account for 50% of the total protein, and 80% of the latter is glutenin [90]. Gliadin exists in the endosperm of corn and is the main storage protein in corn, accounting for about 70% of the total protein of corn [91]. Protein accounts for 10–15% of wheat grains and is an important nutrient in wheat grains. The ultimate value of wheat flour use depends on the properties of seed storage proteins, especially gluten protein.

#### 4.3.1. Factors Affecting Protein Quality in Crops

A reasonable amino acid composition will greatly improve the quality of protein. There are nine amino acids that cannot be synthesized by the human body but must be obtained from the outside world: isoleucine, leucine, lysine, methionine, phenylalanine, threonine, tryptophan, valine, and histidine [92]. When amino acid intake is insufficient, it will have a negative impact on human growth and development. Soy protein contains all essential amino acids, but less methionine and cysteine [93]. 7S co-globulins contain fewer sulfur-containing amino acids, and S-adenosyl methionine deficiency may increase the incidence of chronic liver disease [94]. In addition, the α subunit of β-conglycinin in 7S co-globulins is one of the main factors causing allergy to soy products [95]. Therefore, it is important to improve soybean protein by adjusting the proportion of 7S and 11S globulin components and increasing the methionine content in soybean protein.

Wheat gluten can trigger some diseases in susceptible individuals while ensuring dough quality [96]. The most prevalent disease is celiac disease (CD), an autoimmune response triggered by the immunogenic epitope located on wheat α-gliadin [97]. Currently, the only treatment available is a gluten-free (GF) diet [98]. Therefore, the cultivation of crops with low gliadin protein content is necessary for CD patients.

#### 4.3.2. Gene Resources for Protein Improvement in Rice, Wheat, Corn, and Soybean

The genes encoding 7S co-globulin are a large family with at least 15 members, which are located in different regions of chromosomes and have different degrees of homology. The three subunits in β-companion soy globulins are controlled by *Cgy1*, *Cgy2*, and *Cgy3* respectively [99]. The gene family of 11S globulins has at least five members, *Gy1* to *Gy5* [100]. It is possible to adjust the content of sulfur-containing amino acids and the ratio of 11S/7S in soybean seeds by controlling the genes mentioned above.

Wheat gliadin genes appear in closely linked clusters, called blocks, which exist at six complex chromosome sites (*Gli-A1*, *Gli-B1*, *Gli-D1*, *Gli-A2*, *Gli-B2*, and *Gli-D2*). γ-Gliadin and ω-gliadin are encoded by gene clusters of *Gli-1* loci on the short arm of homologous group 1 chromosome, and α-gliadin is controlled by *Gli-2* loci on the short arm of group 6 chromosomes [101]. At present, five TFs related to zein accumulation in corn have been identified, including O2, PBF1, OHPs, ZmMADS68, and ZmbZIP22, which are all regulatory factors that directly regulate the transcription of the zein gene [102]. However, only two of them (ZmMADS68 and ZmbZIP22) are improved by CRISPR/Cas9 gene editing technology, leading to an improvement in kernel protein quality by reducing contents of zein, which lacks two essential amino acids, lysine and tryptophan [102,103]. By mining the gene resources of rice, wheat, corn, and soybean, a list of genetic resources that can improve protein quality was derived (Table 3). Through the editing of these key genes, crop proteins with more balanced amino acid ratios and wheat suitable for CD patients have been obtained [8,9,97,98,102,103,104,105,106,107,108], suggesting that it is an efficient strategy to improve the protein quality of main crops, by manipulating the related genes based on gene editing.

### 4.4. Identification of Genetic Resources for Other Health Functional Components in Crops

#### 4.4.1. Flavonoids in Crops

Flavonoids are unique secondary metabolites of polyphenols in plants. Flavonoids are usually divided into seven subclasses: flavonols, flavonoids, isoflavones, anthocyanins, flavonoids, flavanols, and chalcones [109]. Rice, corn, wheat, and soybean all contain flavonoids with different compositions and contents. Flavonoids have antioxidant, antibacterial, anti-inflammatory, anti-tumor, cardiovascular disease-preventative, and nerve-protective activities. Currently, research on the biological activity of flavonoids has become an important research topic worldwide.

Isoflavones mainly exist in legumes, usually in the form of glycosides in nature [109]. Soybean isoflavones are also known as phytoestrogens [110], acting as estrogens in the body to prevent osteoporosis and slow down aging through various pathways [111,112]. Studies have shown that soy isoflavones can significantly reduce the risk of cardiovascular disease for premenopausal women [113]. With the increasing demand for soy isoflavones, it becomes very meaningful to increase the content of isoflavones in soy via means of genetic engineering.

Anthocyanin is a kind of water-soluble pigment belonging to phenols, which is unstable in nature [114]. Anthocyanins are the most efficient antioxidants and free radical scavengers that have been discovered so far [115]. They not only inhibit the proliferation of cancer cells and promote the death of cancer cells but also exert a variety of health effects such as anti-inflammation, blood fat-lowering, intestinal health-improving, and nervous system- and retina-protecting [116]. Colored crops have attracted more and more attention from scholars because they contain an abundance of proanthocyanidins and anthocyanins.

#### 4.4.2. Minerals, Vitamins, and Other Active Substances in Crops

Mineral malnutrition is one of the world’s most serious challenges to humanity. It is estimated that there are 150 million children under the age of 4 in the world who do not get enough vitamins and minerals [117]. Mineral malnutrition can be solved by mineral supplementation, food fortification, or increasing the concentration or bioavailability of mineral elements. Vitamin A and carotenoids cannot be synthesized directly by the human body, but must be supplemented from plant (provitamin A carotenoid) or animal (retinol) dietary sources. Vitamin A deficiency increases the risk of child death [118]. α, β, γ-Carotene and β-cryptoxanthin are precursors for the synthesis of vitamin A in humans, which is effective in preventing nyctalopic, gastrointestinal diseases, and measles and improving resistance and may influence the development of obesity, insulin resistance, hepatic steatosis, and cardiovascular diseases [119]. Phytic acid (PA, IP6) is one of the main anti-nutritional components in wheat, rice, soybean, and other crops, and it can chelate important micronutrients to form phytate, prevent the intestinal absorption of Fe and Zn, and limit the bioavailability of trace elements. Therefore, the bio-fortification of crops by reducing the concentration of anti-nutrients and other secondary metabolites can be regarded as an important means to improve the bioavailability of mineral elements [120].

#### 4.4.3. Identification of Gene Resources for Improving Health Functional Components in Crops

Plant flavonoid synthesis is mainly dependent on the phenylalanine pathway, which is influenced by phenylalanine ammonia-lyase (PAL), cinnamate 4-hydroxylase (C4H), 4-coumarate coenzyme A ligase (4CL), chalcone synthase (CHS), chalcone isomerase (CHI), isoflavone synthase (IFS), flavanone-3-hydroxylase (F3H), dihydroflavonol 4-reductase (DFR), anthocyanidin synthase (ANS), and anthocyanidin reductase (ANR) [121]. Besides the enzymes related to flavonoid synthesis, many TFs are also very important for the regulation of flavonoid synthesis, such as MYB [122], the basic helix-loop-helix (bHLH) [11], and WD40 [123]. R2R3-MYB is one of the MYB TFs, which can control the synthesis of flavonoids by changing the expression of genes encoding F3H, CHS, FLS, and CHI [124,125]. The *Rc* gene in rice edits the bHLH transcription factor, and most rice shows a white color due to the lack of some sequences of the *Rc* gene. The anthocyanin content in rice is increased obviously when gene editing technology is used to restore the function of *Rc* [11].

Observations based on low-phytic acid mutants of corn and wheat show that PA synthesis is relatively conservative in most crops. The research shows that PA content decreases to different degrees, and the content of minerals, such as Zn, Fe, P or Ca, increases, after mutation of the ABC transporter and related enzymes involved in PA biosynthesis in corn, rice, and wheat. ZFNs and CRISPR/Cas9 were used to edit the gene *IPK1* encoding the key enzyme in the phytic acid biosynthesis pathway (inositol pentakisphosphate 2-kinase 1) in wheat and corn, respectively. The phytic acid content in the mutant decreased significantly, and the accumulation of Fe and Zn increased in wheat [126,127]. In rice mutants with the phospholipase D gene (OsPLDα1) produced by the CRISPR/Cas9 system, the phytic acid content decreased obviously, which indicated that phospholipase D might participate in phytic acid biosynthesis through a lipid-dependent pathway [128]. Cd is a carcinogenic heavy metal, which can be absorbed by plants through roots, transported to grains, and accumulated in the human body through the food chain, thus damaging human health [75,129]. Several transporter proteins related to Cd accumulation that can be controlled via genetic engineering in rice have been identified in recent years, but the only two of transporter proteins currently regulated by gene editing are OsLCT1 and OsNramp5 [130,131,132].

Through gene mining of rice, wheat, corn, and soybean, a list of gene resources for improving other health functional components of the four crops using gene editing technology was obtained and is shown in Table 4.

## 5. Discussion

As one of the designed breeding technologies, gene editing is of great practical and economic significance for improving the nutritional quality and physiological activity of crops. Because of its accuracy and high efficiency, it is believed that it will bring a revolution to the agricultural section soon [136]. However, in the field of breeding, gene editing technology faces unprecedented challenges along with a broad application prospect.

The regulatory policies, patent monopoly, and social acceptance have large effects on the development of gene-edited crops [137,138]. From the aspect of consumers, the social acceptance of gene-edited crops is higher than that of genetically modified (GM) crops; however, many people now know little about gene-edited crops and may even confuse them with GM crops [139]. Therefore, ways to popularize the safety of gene-edited crops will become a huge challenge for their commercialization. Strengthening the popularization of science based on gene-edited crops through internet media and affixing specific labels to genetically edited products may help to deal with this problem. At present, the attitudes of governments around the world towards gene-edited crops are quite different. Based on the fact that gene-edited crops do not introduce exogenous genes, many experts believe that they are no different from conventionally breed crops, and there is no additional risk to safety [24]. Nowadays, many countries in the European Union think that gene-edited crops should not be equal to GM crops, and they want to relax the supervision of genetically edited crops. Accordingly, some countries have started to investigate whether the regulation of gene-edited crops should be relaxed, and it is expected that more scientific and reasonable policies will be formulated to supervise gene-edited crops in the future.

The development of gene-edited crops is not only facing challenges from policy systems, but also many challenges in technical optimization. At present, the most mature application of gene editing technology is the improvement of single gene traits [140]. Because the synthesis of many components of crops is actually controlled by multiple genes, the optimization of multiple gene editing technology is very important in the field of pyramid breeding. In a polygene editing system, multiple sgRNAs are usually used to achieve the editing of multiple genes; however, the number and type of genes that can be operated simultaneously are limited in this process [141]. Too many target genes will increase the probability of off-target effects, and at the same time, transfection will be difficult because the final plasmid construct is too large. Through the continuous improvement of gene editing technology and editing strategies, researchers look for smaller nucleases with higher specificity, such as Cas12a, and use specific nuclease transport methods to reduce the off-target effect and improve the efficiency of gene editing [142]. In addition, the development of more efficient off-target detection technologies is also one of the important directions to promote the application of gene editing to breeding and accelerate the commercialization of gene-edited crops.

There have been some successful cases of gene editing to improve crop quality. In 2019, the non-GM soybean with high oleic acid based on TALEN editing technology from the Calyxt Company of the United States was successfully marketed after passing the food safety evaluation of the FDA. Recently, Suike No.8, the first non-GM soybean with high oleic acid content, improved via gene editing technology in China, also will enter the safety evaluation stage. In 2021, a company in Japan first debuted a tomato rich in γ -aminobutyric acid, which was improved via CRISPR technology and has the functions of lowering blood pressure and promoting relaxation, and it conceivably has good market demand. Nowadays, many ingredients extracted from plants are added to functional foods and health products, putting forward the need for more functional crops and creating stronger driving forces for the innovation and application of gene editing technology. On the other hand, the improvement of nutritional quality in crops through gene editing technology offers the possibility to alleviate the global food security problem and the pressure based on nutritional needs. Especially, for developing countries, previous studies have shown that the improvement of essential nutrients in crops can greatly improve people’s nutritional health status based on the premise that sufficient food is not available [143].

Because of the differences in national regulatory policies and the development of gene editing technology, the research status in the field of gene editing for improving crop nutrition has demonstrated very large differences worldwide. In terms of commercialization, the monopoly of gene editing tools is the main reason why some countries with relatively lax regulation of gene-edited crops have not yet been able to have commercial gene-edited crops. Although different research groups do not need very close cooperation to improve the nutritional quality of crops via gene editing, food security and malnutrition are global problems. In the future, research groups should accelerate the application of gene editing to improve crops through cooperation and communication.

## 6. Conclusions

This paper analyzed the progress and trends in research on improving crop nutritional quality through gene editing technology by means of a bibliometric analysis. Since 2005, there has been a significant increase in the literature in this field, where the United States is in a leading position, some European countries, such as France and the UK, have high citation volumes, and China performs quite actively. Rice is the most studied crop, and the research on improving the content and quality of protein in crop breeding has occurred earlier than that on other nutrients. After systematic evolution, the research hotspots now focus on the regulatory network of related traits, quite different from the technical improvements in gene editing in the early stage. Then, quality-related genes that can be edited to develop functional crops were mined, including 16 genes related to starch, 15 to lipids, 14 to proteins, and 15 to other functional components. Although the innovation of gene editing technology has developed rapidly, its application was delayed due to regulatory policies, patent monopolies, and social acceptance, which needs cooperation and communication worldwide in terms of technology development and popularization.

## Figures and Tables

**Table 1:** Gene resources of starch biosynthesis and their applications to crop improvement using CRISPR/Cas9 gene editing.

	Gene Names	Gene ID	Encoding Proteins	Phenotype	Functions of Products	References
Wheat	*TaSBEIIa*	TraesCS2A02G293400 TraesCS2D02G290800 TraesCS2B02G309500	starch branching enzyme	Increased amylose content	Foods with high amylose content and RS are helpful to improve human health	[52]
	*TaWaxy*	TraesCS7A02G070100 TraesCS4A02G418200 TraesCS7D01G064300	Granule-bound starch synthase	Lower amylose content	Waxy wheat is formed to improve edible taste	[56]
Corn	*Waxy*	Zm00001eb378140	Granule-bound starch synthase I	Amylopectin content and grain yield of hybrid were significantly increased	Waxy maize produces mainly amylopectin starch with special food or industrial values	[57,58]
	*SHRUNKEN2 (SH2)*	Zm00001d044129	ADP glucose pyrophosphorylase	The amylopectin content with the WX gene (SH2SH2wxwx) exceeds 96%	Fresh corn with super-sweet and waxy compound flavor	[60]
Rice	*NF-YB1*	LOC_Os02g49410	NF-Ys TFs	Higher chalkiness in crnf-yb1 mutants	Regulates rice grain quality (lower starch, higher crude protein contents)	[53]
	*Waxy*	LOC_Os06g04200	granule-bound starch synthase I	Significant decrease in AC in the endosperm; amylose content decreased from 14.6% to 2.6% in the mutants	Improves the eating and cooking quality (ECQ) of rice	[7,59]
	*OsSSIIb*	LOC_Os02g51070	soluble starch synthase II	Decreases the amylose content, gelatinization temperature	Improving the ECQ of rice	[48]
	*SBEIIb*	LOC_Os02g32660	starch branching enzyme IIb	Amylose content is increased, and the RS content is also increased	Beneficial for diabetes and kidney disease patients	[46,47,50,55]
	*MADS78; MADS79*	LOC_Os09g02830; LOC_Os01g74440	MADS box TFs	Loosely packed spherical starch granules	Impact both seed size and quality in rice	[61]
	*OsAAP6; OsAAP10*	LOC_Os01g65670; LOC_Os02g49060	amino acid transporter	Amylose content was down-regulated significantly	Improving ECQ of rice	[62]
	*NF-YC12*	LOC_Os05g23910	NF-Ys TFs	Total starch and amylose contents were significantly lower than those in WT	Increase the grain size and weight in rice	[63]
	*GS9*	LOC_Os09g27590	an unknown protein	Significant decrease in chalkiness	Increase the grain size and weight	[64]
	*ISA1*	LOC_Os08g40930	isoamylase 1	Total starch, amylose, and amylopectin in mutant endosperm were reduced	Beneficial for rice quality breeding	[65]
	*AH2*	LOC_Os09g23200	MYB-domain protein	Enhanced grain quality, including decreased amylose content, and increased protein content	Required for hull epidermis development, palea identity, and grain size	[66]

**Table 2:** Gene resources of lipid biosynthesis and their applications in crop improvement based on gene editing.

	Gene Names	Gene ID	Encoding Proteins	Strategies of Gene Editing	Phenotype	Functions of Products	References
Soybean	*GmFATB1a* *GmFATB1b*	Glyma. 05G012300 Glyma.17G012400	acyl-acyl carrier protein thioesterases	CRISPR/Cas9	Decreased palmitoleic acid and stearic acid contents	Palmitic acid promotes cancer cell metastasis	[82]
	*GmLox1* *GmLox2* *GmLox3*	Glyma.13G347600 Glyma.13G347500 Glyma.15G026300	lipoxygenase isozymes LOX1	CRISPR/Cas9	Lipoxygenase-free soybean	Decreases soybean production and processing cost	[83,84]
	*GmFAD2*	*GmFAD2-1A*: Glyma.10G278000*GmFAD2-2A*: Glyma.19G147300	Δ12-fatty acid desaturase II (FAD2)	CRISPR/Cas9	Oleic acid content is increased and linoleic acid content is decreased significantly	Reduces chemical hydrogenation and reduces processing costs	[79]
	*FAD2-1A FAD2-1B*	Glyma10g42470 Glyma20g24530	fatty acid desaturase 2	TALENs	Oleic acid is increased from 20% to 80% and linoleic acid is decreased from 50% to less than 4%	Healthier oils with high monounsaturated fats and a longer shelf-life	[80]
	*FAD3A* *FAD3B* *FAD3C*	Glyma14g37350 Glyma02g39230 Glyma18g06950	fatty acid desaturase 3	TALENs	Significantly altered fatty acid levels. Linoleic and linolenic acids are decreased to levels below 3%, and oleic acid is increased to levels over 80%	High levels of polyunsaturated linoleic and linolenic acid	[85]
	*Gm15G117700*	/	a regulator of soybean oleic acid synthesis	CRISPR/Cas9	Reduced linoleic acid and increased stearic acid	Improves the quality of soybean fatty acids	[86]
	*FAD2-2*	GLYMA_03G144500	microsomal-6 desaturase	CRISPR/Cas9	Higher oleic acid and decreased palmitic acid	Decreases the risk of type diabetes	[81]
Rice	*OsFAD2-1*	LOC_Os02g48560	enzyme fatty acid desaturase 2	CRISPR/Cas9	Increased oleic acid and decreased linoleic acid	Suppresses lifestyle diseases	[77]
	*OsMGD2*	LOC_Os02g55910	Monogalactosyldiacylglycero synthase	CRISPR/Cas9	Decreased linoleic acid content (26.6%)	Improves plant growth and grain quality	[87]

**Table 3:** Gene resources for protein improvement in rice, wheat, corn and soybean.

	Gene Names	Gene ID	Encoding Proteins	Strategies of Gene Editing	Phenotype	Functions of Products	References
Soybean	/	Glyma.20g14840 Glyma.03g163500 Glyma.19g164900	conglycinins (7S) and glycinins (11S)	CRISPR/Cas9	Adjusting the ratio of 7S and 11S globulin components	Increases the sulfur amino acid content in the protein	[9]
Corn	*ZmbZIP22*	*Zm00001eb318740*	bZIP-transcription factor 22	CRISPR/Cas9	27 kD γ-zein transcript levels are reduced to ~70% in the mutants	Improves kernel protein quality by reducing zein contents	[102]
	*ZmMADS*	*Zm00001eb006480*	MADS-transcription factor 68	CRISPR/Cas9	Zeins are significantly decreased (12.5%) in MADS/CAS9-21 kernels	/	[103]
Wheat	*Gli-2*	TraesCS6B02G065800	alpha-gliadin	CRISPR/Cas9	Decreases alpha-gliadins and promotes a compensatory effect on glutenin	Associated with the development of celiac disease and non-celiac gluten sensitivity	[97]
	*TaASN2*	TraesCS3A02G077100	asparagine synthetase	CRISPR/Cas9	Reduces free asparagine in the grain	Free asparagine is the precursor for acrylamide formation	[104]
	*TaATI*	LOC543286 LOC543281	alpha-amylase/trypsin inhibitor	CRISPR/Cas9	Decreases alpha-amylase/trypsin inhibitor content	Involved in the onset of wheat allergies and non-celiac wheat sensitivity	[105]
	*Gli-2* *Gli-1*	TraesCS6A02G049100 TraesCS1A02G007400	alpha-gliadin gamma-gliadin	CRISPR/Cas9	Decreases alpha-gliadin Decreases gamma-gliadin	Provides bread and durum wheat lines with reduced immunoreactivity for gluten nutrition consumers	[98]
	*TaNAC019-A* *TaNAC019-B* *TaNAC019-D*	TraesCS3A02G077900 TraesCS3B02G092800 TraesCS3D02G078500	endosperm-specific NAC transcription factor	CRISPR/Cas9	Leads to lower gluten and starch contents	Improves grain yield and quality, changing the dough strength and quality	[106]
	*TaPDI*	LOC100037546 LOC542857 LOC542858	wheat protein disulfide isomerase	CRISPR/Cas9	Glutenin macropolymer (GMP) content is decreased significantly	Low-gluten dough is good for celiac disease patients	[107]
Rice	*qSP3*	LOC_Os03g57960	a cupin domain protein for the synthesis of 52 kDa globulin	CRISPR/Cas9	Lack of accumulation of 52 kDa globulin	Improves seed vigor in rice, and significantly lower amino acid contents were observed in the mature grains	[108]
	*GW2*	LOC_Os02g14720	RING-type E3 ubiquitin ligase	CRISPR/Cas9	Higher grain protein content and accumulation of essential dietary minerals in the endosperm	Improves grain architecture and grain nutritional quality and is an important modulator of plant morphology	[8]

**Table 4:** List of gene resources of other health functional components in rice, wheat, corn, and soybean improved by gene editing.

	Ingredient	Gene Names	Gene ID	Encoding Proteins	Strategies	Phenotype	Functions of Products	References
Wheat	Mineral	*TaIPK1*	TraesCS6A02G139400	inositol pentakisphosphate kinase	CRISPR/Cas9	Decreased phytic acid content and enhanced iron and zinc content in wheat grains	Contributes to the development of biologically fortified wheat and reduces malnutrition	[126]
Rice	Mineral	*OsPLDa1*	LOC_Os01g07760	phospholipase D	CRISPR/Cas9	Depletion of phosphatidic acid production and significantly reduced phytic acid content	Improves the cooking and eating quality and nourishment of brown rice	[128]
	Mineral	*OsLCT1*;	LOC_Os06g38120	a rice homolog of wheat low-affinity cation transporter 1	CRISPR/Cas9	Low grain Cd accumulation	Rice can be safely produced in Cd-polluted soil	[130]
	Mineral	*OsNramp5*	LOC_Os07g15370	natural resistance-associated macrophage protein 5	CRISPR/Cas9	Significantly reduced accumulation of Cd in the grains. However, the degree of OsNramp5 mutation will affect the yield	Rice can be safely produced in Cd-polluted soil	[130,131,132]
	Anthocyanin	*Rc*	LOC_Os07g11020	bHLH transcription factor	CRISPR/Cas9	Increased proanthocyanidins and anthocyanidins in the mutants	Promotes health	[11]
	Anthocyanin	*OsTTG1*	LOC_Os02g45810	WD40 repeat protein	CRISPR/Cas9	Almost no anthocyanin accumulation in the leaf tip and ear	OsTTG1 is an important regulator of anthocyanin biosynthesis	[133]
	γ -aminobutyric acid	*OsGAD3*	Os03g0236200	glutamate decarboxylase 3	CRISPR/Cas9	Seven-fold higher GABA content and significantly higher grain weight and protein content	Improves life-style related diseases, such as hypertension, diabetes, and hyperlipidemia	[134]
	Flavonoid	*OsCOP1*	LOC_Os02g53140	constitute photomorphosis 1	CRISPR/Cas9	Flavonoid accumulation in *yel-hc* mutant is reduced in the embryo and endosperm	Participates in embryo development and flavonoid biosynthesis in rice grains	[123]
Corn	Mineral	*IPK1*	Zm00001eb067500 Zm00001eb432760	inositol pentakisphosphate kinase	ZFNs	The level of phosphoric acid decreases, and inorganic phosphate increases	Provides a basis for the development of reduced phytic acid traits with ecological significance	[127]
	Anthocyanin	*SWEET13a SWEET13b SWEET13c*	Zm00001eb408920 Zm00001eb408900 Zm00001eb133100	sucrose transporter	CRISPR/Cas9	Triple knock-out mutants are stunted, showing severe chlorosis of all leaves	With extreme chlorosis, massive anthocyanin accumulation	[135]
Soybean	Isoflavone	*GmF3H1, GmF3H2 GmFNSII-1*	Glyma.02G048400 Glyma.02G048600 Glyma.12G067000	flavanone-3-hydroxylase (F3H) and flavone synthase II (FNS II)	CRISPR/Cas9	Homozygous triple mutants have approximately twice the leaf isoflavone content	Isoflavones reduce the incidence of specific cancers and improve cardiovascular disease risk markers	[125]

## Data Availability

Not applicable.

## Supplementary Materials

The following supporting information can be downloaded at: https://www.mdpi.com/article/10.3390/nu15020373/s1, Figure S1: Relationship between the activity and influence of the top 11 countries in terms of publication volume; Figure S2: Burst map of keywords from 2005 to 2022; Table S1: Search strategy for investigating crop improvement through gene editing technology in the Web of Science.
