# Supplementary material for: Bibliometric Analysis of Functional Crops and Nutritional Quality: Identification of Gene Resources to Improve Crop Nutritional Quality through Gene Editing Technology
Source: Nutrients. 2023 Jan 11;15(2):373. doi: 10.3390/nu15020373 (PMC9865409; doi:10.3390/nu15020373)
Supplement: Supplementary file 1 [file nutrients-15-00373-s001.zip › nutrients-2103933-supplementary.pdf]

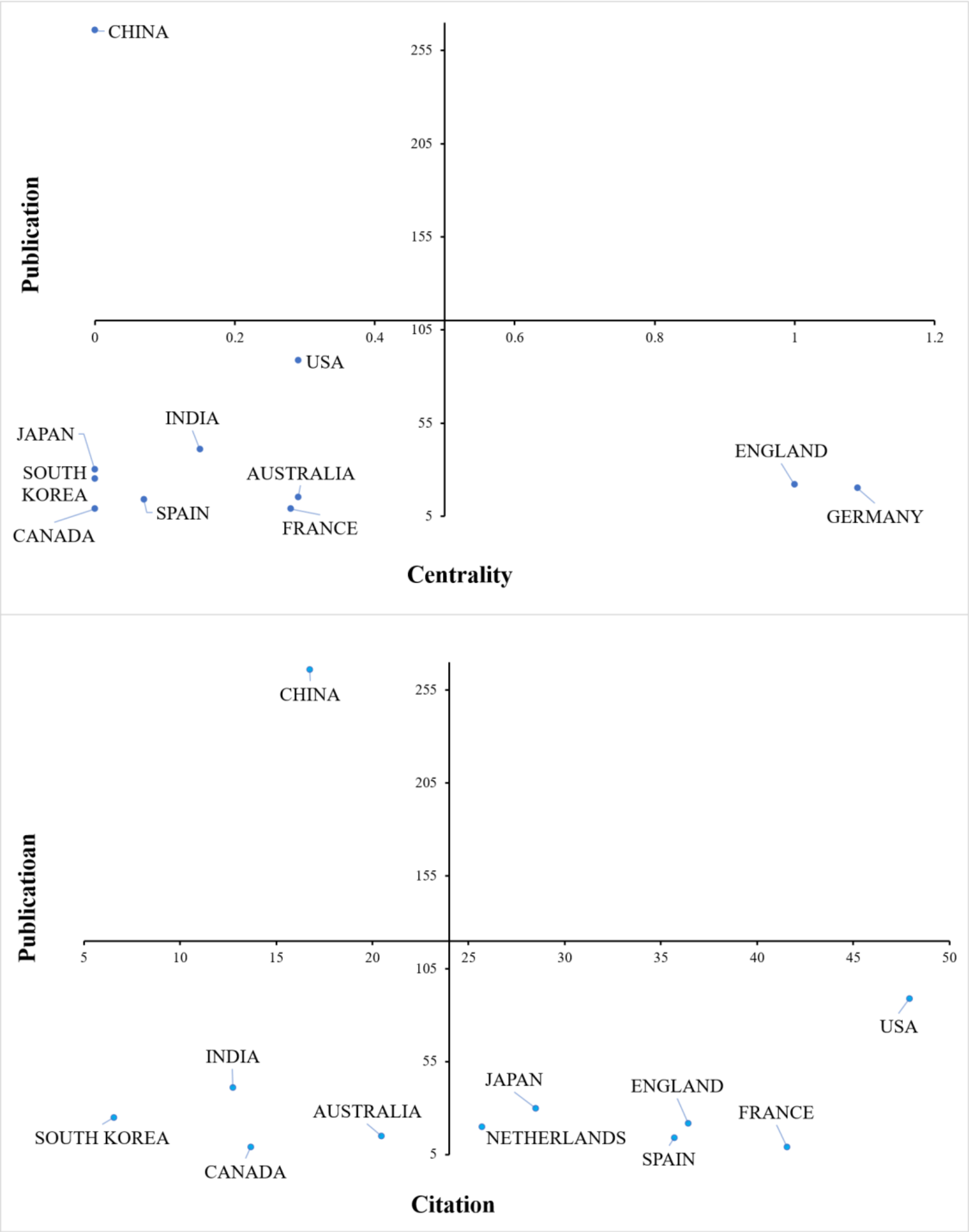

Supplementary Figure S1. The relationship between the activity and influence of top 11 countries in terms of publication volume. (A) The relationship between the number of national volume and centrality of the publication（B）The relationship between the number of national volume and total citations of the publication.

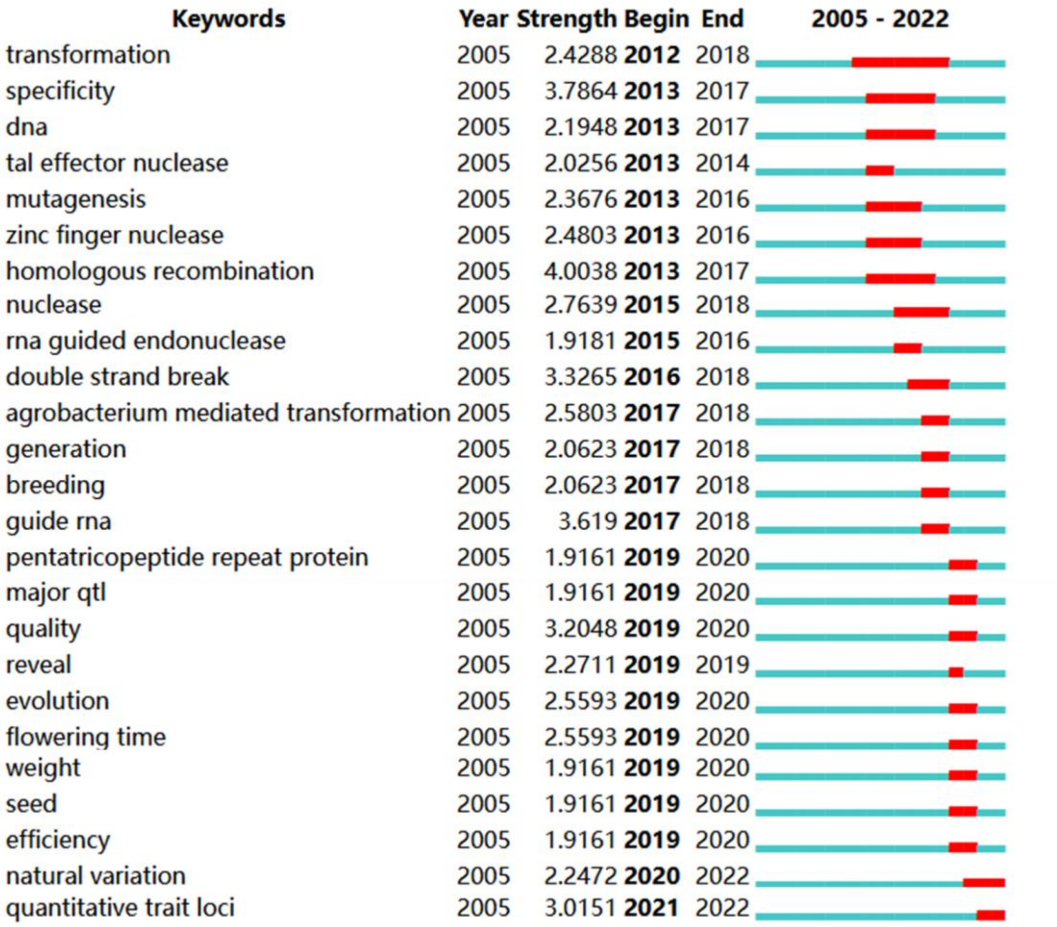

Supplementary Figure S2. Burst map of keywords from 2005 to 2022

Supplementary Table S1. Search strategy for investigating crop improvement through gene editing technology in the Web of Science (updated September. 24, 2022)

Component	Search Strategy
**Gene editing technology**	**TS= ("zinc finger nucleas*" OR "Zinc finger endonuclease*" OR "zinc-finger nucleas" OR "TALE nuclease" OR "TALEN" OR TALN OR TALENS OR TALNS OR "Transcription activator-like effector*" OR "Transcription activator-like nuclease*" OR "TAL effector nucleases" OR "TAL nuclease*" OR "clustered regularly interspaced short palindromic repeat*" OR "CRISPR*" OR "RNA-Guided Cas9" OR "effector nucleases" OR "genome editing" OR "gene editing" OR "engineered endonuclease*" OR "Engineered nuclease*" OR "site-directed nuclease*" OR "genome engineering" OR "chimeric nucleases" OR "Targeted modification" OR "Sequence-Specific Nucleases" OR " base editing" OR "Multiplex gene editing" OR "Cas9-guide RNA ribonucleoprotein " OR RNP OR "prime**

	**editing guide RNA" OR pegRNA OR "Prime editors" OR PEs)**
**Various crops**	**TI=(crop or "oryza sativa" or "oryza glaberrima" or rice or wheat or "Triticum aestivum" or "taestivum" or "Secale cereal*" or rye or Thinopyrum or "Lophopyrum elongatum" or "Agropyron elongatum" or maize or maizes or "Zea mays*" or corn or soybean or soybeans or "soy bean" or "soy beans" or soyabean or soyabeans or "soya bean" or "soya beans" or "Glycine max" or "Glycine soja" or soy or soys or soya or soyas)**
**main nutrient components**	**TS=(nutrition or nutrient or quality or protein or lysine Or tryptophan or “Amino Acid” or methionine or starch or Amylose Or amylopectin or “oil content” or lipid or vitamin or mineral or “Phytic acid” or “Anthocyanin” or “ Aromatic compound” or polyunsaturated or monounsaturated or “fatty acid” or oleic or linolenic or isoflavone or “omega-6” or “phenolic acids” or biofortification) Not TS=(resistance or “Male sterility” or tolenrance or stress or insect or virus)**

TS=Abstract and Keywords; TI=Title; Indexes=SCI-EXPANDED
